# Study motivation and academic performance among first-year five-year junior college nursing students: a mixed-methods study

**DOI:** 10.1186/s12912-026-04460-y

**Published:** 2026-03-02

**Authors:** Mei-Hsiu Lee, Szu-Mei Hsiao, Chin-Chin Hsu

**Affiliations:** 1 Department of Nursing, MeiHo University, No. 23, Pingguang Rd., Neipu Township, Pingtung, 912009 Taiwan; 2 Department of Nursing, National Chi Nan University, Nantou, Taiwan; 3 Department of Nanzih Junior High School, Kaohsiung, Taiwan

**Keywords:** Nursing students, Study motivation, Academic performance, Mixed-methods research

## Abstract

**Background:**

The shortage of nursing professionals in Taiwan has become an urgent public health concern, affecting healthcare quality and workforce sustainability. Graduates of five-year junior college nursing programs represent a major source of entry-level nurses. Understanding the learning motivations and professional perceptions of newly admitted students is therefore essential for strengthening nursing education and supporting early workforce retention. This study aimed to explore first-year students’ learning motivations, perceptions of the nursing profession, and the relationships between motivation and academic performance.

**Methods:**

This explanatory sequential mixed-methods study employed a cross-sectional quantitative survey followed by qualitative focus group interviews. A total of 93 first-year students (aged 15–16 years; predominantly female) enrolled in a five-year junior college nursing program in southern Taiwan completed the Diverse Motivations for Studying Nursing (DMSN) scale, a 46-item instrument rated on a five-point Likert scale that assesses intrinsic and extrinsic motivations for studying nursing. Academic performance data, including grades in Introduction to Nursing, First Aid, and overall semester grade point average (GPA), were obtained from academic records. Quantitative data were analyzed using independent-sample t-tests and logistic regression. In addition, focus group interviews were conducted with 24 students, and qualitative data were analyzed using clinical qualitative content analysis to further explain quantitative findings.

**Results:**

Quantitative analysis showed that extrinsic motivation significantly predicted students’ performance in Introduction to Nursing (OR = 1.073, *p* = .018), whereas intrinsic motivation was not a significant predictor of academic outcomes. No significant associations were found between motivation and performance in First Aid or overall semester GPA. Qualitative analysis identified five major motivations for studying nursing: choosing courses of interest, the desire to use professional knowledge to help others, job stability, obtaining a nursing license, and viewing nursing as a calling. Students perceived nursing as a demanding profession requiring responsibility, empathy, and strong professional competence.

**Conclusions:**

Extrinsic motivation plays a key role in academic performance during the early stage of nursing education. Nursing educators should incorporate motivation-oriented strategies, career-related guidance, and professional identity development into first-year curricula to enhance student engagement and support future workforce retention.

**Supplementary Information:**

The online version contains supplementary material available at 10.1186/s12912-026-04460-y.

## Introduction

Taiwan is currently facing a severe shortage of nursing manpower. The National Union of Nurses Associations of the Republic of China has warned that this shortage has led to nationwide emergency department overcrowding, threatening not only the healthcare system but also national security (National Union of Nurses Associations of the Republic of China, 2025) [1]. In recent years, continuous attrition of nursing personnel has placed considerable strain on hospital staffing, with some institutions forced to close wards due to insufficient manpower [2]. Difficulties in retaining newly graduated nurses, combined with an aging nursing workforce, have further reduced the availability of care for individuals in need, raising concerns about social and healthcare security. Graduates from five-year junior college nursing programs remain one of the primary sources of entry-level clinical nurses in Taiwan. Early learning motivation and academic performance have been shown to influence nursing students’ professional identity development, persistence in training, and intentions to remain in the profession. Understanding motivation and academic engagement at the initial stage of nursing education may therefore provide critical insights into long-term workforce retention.

Nursing is both an art and a science, encompassing the protection and promotion of health, the prevention of illness and injury, the facilitation of recovery, and the alleviation of suffering [3]. Nurses are educated to practice based on specialized knowledge, professional values, and clinical skills that define the nursing profession [4]. Despite the essential role of nursing, workforce shortages persist, and nursing continues to be a female-dominated profession, with women significantly outnumbering men among nursing students and practitioners [5].

Motivation plays a critical role in students’ learning processes and professional development. According to Self-Determination Theory (SDT), individuals possess inherent psychological resources that support motivation, personality development, and behavioral regulation [6]. Motivation is a dynamic construct that influences behavior across personal and professional contexts and is closely linked to learning engagement and academic performance [7]. In educational settings, insufficient vocational motivation has been associated with reduced learning efficiency and increased burnout [8]. For nursing students, motivation is particularly relevant, as the decision to study nursing involves both personal aspirations and external influences.

Intrinsic and extrinsic motivation represent two key dimensions of motivation. Intrinsic motivation refers to engagement driven by interest, enjoyment, and the inherent satisfaction of learning, whereas extrinsic motivation involves behavior oriented toward external rewards or social expectations [6, 7]. Although individuals are naturally inclined toward intrinsic motivation, educational and social environments can either support or undermine it. Prior research has shown that nursing students’ motivations for entering the profession are often associated with altruistic values, family encouragement, and perceived employment opportunities [9, 10]. Motivation has also been identified as a key factor influencing learning outcomes and student retention in health professions education [11, 7].

Professional identity development is a central task during adolescence and early adulthood. Among nursing students, positive perceptions of the profession and strong learning motivation have been associated with professional identity formation, career satisfaction, and confidence in entering the nursing workforce [12, 13]. Students’ attitudes, expectations, and concerns further influence their learning capacity and engagement with nursing education [14]. Understanding how newly admitted nursing students perceive nursing and what motivates them to learn is therefore essential for strengthening professional identity and supporting workforce sustainability.

Accordingly, this study aimed to explore the learning motivations and professional perceptions of newly admitted students in five-year junior college nursing programs.

In addition, it examined the relationships among learning motivation, nursing-related academic performance, and semester grade point averages.

To operationalize these aims, this study addressed the following research questions:

1. What are the learning motivations of first-year students in five-year junior college nursing programs?
2. What are these students’ perceptions of nursing as a profession?
3. How do students’ learning motivations relate to their performance in nursing-related subjects and overall semester averages?

## Methods

### Study design

This study employed a cross-sectional mixed-methods design with an explanatory sequential approach. Quantitative data were collected and analyzed first to examine the distribution of study motivation and its relationship with academic performance. Qualitative data were subsequently gathered through focus group interviews to further explain and enrich the quantitative findings. Methodological triangulation was applied to enhance the comprehensiveness and credibility of the results. Among the 93 students who completed the quantitative survey, a subset of 24 students from the same cohort voluntarily participated in the focus group interviews. The qualitative phase was conducted after completion of the preliminary quantitative analysis, following an explanatory sequential mixed-methods design. Quantitative data were collected between November 25 and December 1, 2024. Focus group interviews were conducted between December 10 and December 13, 2024, after the preliminary quantitative analyses were completed. Participants in the qualitative phase were recruited from the same cohort, and separate informed consent was obtained from those who agreed to participate in interviews.

### Study setting

The study was conducted at a university of science and technology in southern Taiwan that offers a five-year junior college nursing program. Data collection took place between November 25 and December 13, 2024.

### Study population and eligibility criteria

The study population consisted of first-year students enrolled in a five-year junior college nursing program at a university of science and technology in southern Taiwan.

Inclusion criteria were enrollment as a first-year student in the five-year junior college nursing program and age between 15 and 16 years at the time of data collection. 

Exclusion criteria were students not enrolled in the nursing program and students with incomplete academic performance records for the target courses. 

Participation was voluntary. Issues related to informed consent are described in the Ethical Approval section.

### Sample size and sampling technique

For the quantitative component, total population sampling was adopted to maximize coverage of the target cohort. Among 127 eligible first-year nursing students, 93 completed the questionnaire, yielding a response rate of 73.2%. Because the entire accessible population was targeted, no formal sample size calculation was performed.

For the qualitative component, purposive sampling was used to recruit students who were willing to share their learning experiences and perceptions of the nursing profession. A total of 24 students participated in focus group interviews. Recruitment continued until data saturation was reached, defined as the point at which no new themes or insights emerged from successive interviews.

### Data collection techniques and tools

#### Quantitative data collection

Study motivation was measured using the Diverse Motivations for Studying Nursing (DMSN) scale developed by Lin (1995) [15]. The DMSN consists of 46 items rated on a five-point Likert scale (1 = strongly disagree to 5 = strongly agree) and includes eight factors: Social Relationships, Pursuit of Knowledge, Professional Values and Skills, Career Advancement, Attainment of Status, Enthusiasm, Personal Values, and National and Social Contribution. Reported construct validity ranged from 0.47 to 0.80, and internal consistency reliability (Cronbach’s α) ranged from 0.79 to 0.90. The original Chinese version of the DMSN was used without modification. The internal consistency of the intrinsic motivation construct, calculated based on its four subscales, was excellent (Cronbach’s α = 0.93). The internal consistency of the extrinsic motivation construct, calculated based on its four subscales, was good (Cronbach’s α = 0.84).

Academic performance data, including grades in *Introduction to Nursing*, *First Aid*, and overall semester grade point average (GPA), were obtained with participants’ consent from academic records. Questionnaire data were collected online using the Zuvio platform.

#### Qualitative data collection

This approach is commonly used in qualitative health research to capture diverse perspectives. Qualitative data were collected through semi-structured focus group interviews conducted in a library discussion room. The guiding questions included: “Why did you choose to study nursing?” and “What are your perceptions of nursing work?”

Interviews were audio-recorded, transcribed verbatim within 24 h, and anonymized to ensure confidentiality. An English translation of the interview guide is provided as Additional file 1.

### Data analysis

#### Quantitative analysis

Descriptive statistics were used to summarize participants’ demographic characteristics and motivation scores. Academic performance outcomes included grades in Introduction to Nursing, First Aid, and overall semester grade point average (GPA).

Because the distributions of academic performance variables deviated from normality, median values were used to dichotomize students into higher- and lower-achievement groups for each course and overall semester performance. Independent-sample t-tests were then conducted to compare motivation scores between these groups.

For regression analyses, motivation variables were aggregated into intrinsic motivation (Pursuit of Knowledge, Enthusiasm, Personal Values, and Professional Values and Skills) and extrinsic motivation (Social Relationships, Career Advancement, Attainment of Status, and National and Social Contribution) based on the theoretical framework of Self-Determination Theory and the factor structure of the DMSN scale. According to Self-Determination Theory, motivations oriented toward socially endorsed goals or external expectations are conceptualized as forms of extrinsic motivation. Although the *National and Social Contribution* subscale reflects prosocial values, it emphasizes fulfilling perceived social roles and contributing to society rather than engaging in learning for inherent enjoyment. Therefore, this subscale was classified as extrinsic motivation, consistent with the SDT framework and the original conceptualization of the DMSN scale. Composite scores were calculated by summing the corresponding subscale scores. Although dichotomization using median splits may reduce statistical power, this approach was adopted to enhance interpretability within an educational context, where distinguishing between higher- and lower-achievement groups is meaningful for pedagogical interpretation. Nevertheless, this should be interpreted with caution, and future studies may benefit from modeling academic performance as continuous variables.

Binary logistic regression analyses were performed to examine whether intrinsic and extrinsic motivation predicted academic performance outcomes. Model adequacy was evaluated using omnibus tests of model coefficients, − 2 log likelihood values, Cox and Snell R² and Nagelkerke R² statistics, and the Hosmer–Lemeshow goodness-of-fit test. Odds ratios (ORs) with 95% confidence intervals (CIs) were reported. All statistical analyses were conducted using IBM SPSS Statistics version 23.0 (IBM Corp., Armonk, NY, USA), and statistical significance was set at *p* < .05. Although effect sizes such as Cohen’s *d* were not reported for group comparisons in Table 1, the primary focus of the present analysis was on identifying statistically significant differences in motivation scores. Future studies are encouraged to report effect sizes to facilitate interpretation of the practical significance of observed group differences.

#### Qualitative analysis

Interviews were audio-recorded (not videotaped), transcribed verbatim within 24 h, and coded to ensure confidentiality. Recruitment ceased when data saturation was reached. Following Lincoln and Guba’s criteria for trustworthiness, credibility, confirmability, dependability, and transferability were ensured through member checks, detailed documentation of data collection and analysis, and independent analysis by multiple researchers [16]. Transferability was ensured by providing socio-demographic descriptions of participants and a detailed description of the research process and results, allowing readers to determine whether findings might be applied to other contexts [17]. After transcription, participants reviewed their transcripts for accuracy. Immediately after each session, the researcher completed a reflective journal. All team members independently analyzed the data and compared findings. Qualitative analysis followed the Clinical-Qualitative Content Analysis approach [18], consisting of the following steps: (1) editing and preparing the data for analysis; (2) immersive reading to understand participants’ lived experiences; (3) defining units of analysis; (4) coding for meaning; (5) refining codes and constructing categories; (6) identifying overarching themes; and (7) ensuring methodological rigor through team reflection and validation of analytical decisions. The semi-structured interview guide (English translation) is provided as Additional file 1.

### Ethical approval

Ethical approval was obtained from the Antai Hospital Institutional Review Board (IRB No. 24-113-B; approval date: November 22, 2024). Participation was voluntary, and students were informed that they could withdraw at any time without academic consequences. Written informed consent was obtained from all participating students and their parents or legal guardians prior to data collection. This study was conducted in accordance with the Declaration of Helsinki. 

## Results

### Quantitative findings

#### Participant characteristics

A total of 93 first-year students enrolled in a five-year junior college nursing program participated in the quantitative survey. Participants were aged 15–16 years and were predominantly female. Academic performance data included grades in Introduction to Nursing, First Aid, and overall semester grade point average (GPA).

#### Descriptive results of study motivation

Descriptive statistics for the Diverse Motivations for Studying Nursing (DMSN) scale are presented in Table 1. Among the eight motivational factors, students reported the highest mean scores for Career Advancement, followed by Professional Values and Skills and Personal Values.

Career Advancement reflected students’ perceptions that studying nursing would enhance their employment opportunities, provide stable income, and improve future career prospects. Professional Values and Skills highlighted students’ enjoyment of caring for others and their belief in acquiring correct nursing knowledge and skills to relieve others’ suffering. Personal Values indicated students’ desire to help others, develop patience, and contribute to society.

Although the Attainment of Status factor showed a comparatively lower overall mean score, one item—studying nursing to pass the professional certification examination—demonstrated a relatively high mean score (*M* = 3.55 on a five-point scale), indicating the importance students placed on obtaining professional credentials.

#### Group comparisons by academic performance

Median values for academic performance were 81 for Introduction to Nursing, 83 for First Aid, and 77 for overall semester GPA. Based on these median splits, independent-sample *t*-tests were conducted to compare motivation scores between higher- and lower-achievement groups (Table 1).

As shown in Table 1, all eight motivational factors demonstrated significant differences between achievement groups in First Aid, with higher-achieving students reporting significantly stronger motivation across both intrinsic and extrinsic domains. In contrast, for Introduction to Nursing and overall semester GPA, significant differences were observed primarily for Attainment of Status, indicating that students with higher performance levels reported stronger motivation related to professional recognition and certification.

#### Logistic regression analysis

Binary logistic regression analyses were conducted to examine whether intrinsic and extrinsic motivation predicted academic performance outcomes (Table 2). Extrinsic motivation significantly predicted performance in Introduction to Nursing (*p* = .018; OR = 1.073, 95% CI = 1.012–1.137), indicating that higher levels of extrinsic motivation were associated with increased odds of achieving higher academic performance in this subject. Intrinsic motivation did not significantly predict performance in Introduction to Nursing. The Hosmer–Lemeshow test indicated good model fit, χ²(8) = 5.68, *p* = .683.

Neither intrinsic nor extrinsic motivation significantly predicted performance in First Aid or overall semester.

**Table 1:** Independent samples t-tests of motivation factors by academic achievement groups

Motivation Group	Variable	Introduction to Nursing			First Aid			Semester GPA		
		≤81 / ≥81			≤83 / ≥83			≤77 / ≥77		
		Mean + SD	Mean + SD	t	Mean + SD	Mean + SD	t	Mean + SD	Mean + SD	t
N(%)		45(48.4%)	48(51.6%)		45(48.4%)	48(51.6%)		44(47.35)	49(52.7%)	
Intrinsic Motivation	Pursuit of Knowledge	21.00 + 5.73	22.56 + 6.17	. 210	19.78 + 5.94	23.71 + 5.42	<. 001**	20.73 + 6.02	22.78 + 5.84	. 099
	Enthusiasm	17.27 + 4.89	18.15 + 4.62	. 375	16.44 + 4.74	18.92 + 4.47	. 011*	16.93 + 4.52	18.43 + 4.87	. 129
	Personal Values	12.44 + 3.48	12.96 + 3.61	. 487	11.91 + 3.66	13.46 + 3.29	. 037*	12.09 + 3.37	13.27 + 3.63	. 111
	Professional Values and Skills	16.87 + 4.63	16.90 + 4.08	. 974	15.64 + 4.49	18.04 + 3.87	. 007*	16.23 + 4.37	17.47 + 4.25	. 169
Extrinsic Motivation	Social Relationships	22.13 + 6.00	24.25 + 6.87	. 118	21.60 + 5.93	24.75 + 6.73	. 019*	22.82 + 6.73	23.59 + 6.36	. 570
	Career Advancement	23.87 + 6.52	26.23 + 6.76	. 090	23.02 + 6.85	27.02 + 6.03	. 004*	23.82 + 7.21	26.22 + 6.09	. 084
	Attainment of Status	17.02 + 3.56	19.25 + 3.58	. 003*	16.87 + 3.45	19.40 + 3.58	<. 001**	17.36 + 3.81	18.90 + 3.53	. 047*
	National and Social Contribution	9.22 + 2.70	9.63 + 2.98	. 498	8.73 + 2.78	10.08 + 2.77	. 021*	9.07 + 3.01	9.76 + 2.67	. 247

Note. *p* < .05*, *p* < .01**, *p* < .001***

As shown in Table 2, logistic regression indicated that extrinsic motivation significantly predicted students’ performance in Introduction to Nursing (*p* = .018; OR = 1.073, 95% CI: 1.012–1.137), whereas intrinsic motivation did not.

**Table 2:** Binary logistic regression analysis of intrinsic and extrinsic motivation predicting academic performance

Subject / Variable	*p* Value	Exp(B)	95% CI Lower	95% CI Upper
**Introduction to Nursing**				
Intrinsic motivation	0.08	0.95	0.90	1.01
Extrinsic motivation	0.02*	1.07	1.01	1.14
**First Aid**				
Intrinsic motivation	0.59	1.01	0.96	1.07
Extrinsic motivation	0.22	1.03	0.98	1.09
**Semester Grade Average**				
Intrinsic motivation	0.46	1.02	0.97	1.07
Extrinsic motivation	0.89	1.00	0.96	1.05

Note. Exp(B) = odds ratio; CI = confidence interval. *p* < .05

To further identify which components of extrinsic motivation contributed to this effect, a multiple linear regression analysis was conducted to examine the predictive roles of the four extrinsic motivation subscales on academic performance in Introduction to Nursing. The overall model was statistically significant, F(4, 88) = 3.12, *p* = .019, explaining 8.4% of the variance in course grades (adjusted R² = 0.084).

Among the four extrinsic motivation subscales, attainment of status emerged as a significant predictor of academic performance (β = 0.38, *p* = .012), whereas career advancement, social relationships, and national and social contribution were not significant predictors.

### Qualitative findings

A total of 24 students participated in the focus group interviews. Participants were predominantly female (*n* = 23, 95.8%), with one male student (*n* = 1, 4.2%). Most participants were aged 15 years (*n* = 16, 66.7%), and the remainder were aged 16 years (*n* = 7, 29.2%). All were first-year students enrolled in a five-year junior college nursing program. Detailed demographic characteristics are presented in Table 3.

**Table 3:** Demographic characteristics of focus group participants (*n* = 24)

Characteristic	*n* (%)
**Gender**	
Male	1 (4.2)
Female	23 (95.8)
**Age (years)**	
15	16 (66.7)
16	7 (29.2)

Note. All participants were first-year students enrolled in a five-year junior college nursing program

The qualitative analysis identified five major motivational themes for studying nursing:

4. choosing courses of interest; the desire to use professional knowledge to help others; job stability; obtaining a nursing license, and viewing nursing as a calling.

In addition, students’ perceptions of the nursing profession were categorized into three themes: being busy; having a strong sense of responsibility; and demonstrating empathy.

An overview of the themes and subthemes is presented in Tables 4 and 5.

**Table 4:** Students’ motivations for studying nursing

Main Theme	Subthemes
Choosing courses of interest	Interest in nursing
	Not interested in high school or vocational school courses
	Family recommendation
Desire to use professional knowledge to help others	Increasing medical knowledge
	Helping others
Job stability	Multiple employment opportunities
	Stable income
Nursing is a calling	Belief that nursing embodies noble ideals
Obtaining a nursing license	Gaining the title and professional status of a registered nurse

**Table 5:** Students’ perceptions of nursing

Main Theme	Subthemes
Busy	Irregular day–night shifts
	Exhausting work
	High stress tolerance required
Sense of responsibility	Professional and technical competence
	Observing Conditions and Understanding Needs
Empathy	Being Gentle and Patient
	Being Attentive

### Students’ motivations for studying nursing

Students described multiple reasons for choosing nursing as their field of study. These motivations reflected personal interests, external influences, and pragmatic considerations related to future employment and professional identity.

#### Choosing courses of interest

Some students reported selecting nursing because it aligned with their personal interests and learning preferences. They indicated that they were less attracted to general high school or other vocational pathways and perceived nursing education as more meaningful and engaging.

#### Interest in nursing

Several students expressed genuine interest in nursing work and satisfaction derived from helping others.

I’m interested in nursing work. I think helping people is quite rewarding and gives me a sense of accomplishment. (DA1)

##### Lack of interest in high school or vocational courses

Others stated that they were uncertain about their interests in traditional high school subjects and therefore chose nursing as a clearer and more suitable pathway.

*If I went to high school*,* I’d probably fail many subjects. I’m not very interested in other vocational programs.* (CA2)

##### Family recommendation

Family influence also played a role in students’ decisions. Some participants were encouraged by parents or relatives who viewed nursing as a stable and respectable profession.

*My parents wanted me to study nursing… they believed nursing had good career prospects.* (FA3)

#### Desire to use professional knowledge to help others

Many students described a strong motivation to acquire nursing knowledge and skills in order to help others, particularly family members or vulnerable individuals.

##### Increasing medical knowledge

Students expressed a desire to learn medical knowledge that could be applied in daily life.

*I want to increase my medical knowledge so that I can take care of my family.* (XB1)

##### Helping others

Helping patients and supporting recovery were frequently mentioned as meaningful aspects of nursing.

*Even if patients cannot fully recover*,* I can at least help them get better.* (EB2)

#### Job stability

Pragmatic considerations related to employment were also prominent.

##### Multiple employment opportunities

Students perceived nursing as offering diverse career pathways across healthcare settings.

*Nursing offers various career options.* (AC1)

##### Stable income

Financial security was viewed as an important benefit of the profession.

*It’s easier to find a job*,* and the salary is more stable.* (FC2)

#### Nursing as a calling

A small number of students described nursing as a long-held aspiration and a profession aligned with their personal values.

##### Belief that nursing embodies noble ideals

These students viewed caring for others as a meaningful life purpose.

*I’ve always wanted to be a nurse… getting a license will allow me to help more people.* (JE1)

#### Obtaining a nursing license

Nearly all students emphasized the importance of obtaining a professional license as a prerequisite for practicing nursing.

##### Gaining professional status

Passing the national licensing examination was regarded as essential for recognition as a registered nurse.

*Since I’m studying nursing*,* I have to pass the licensing exam. Only then can I work and care for patients.* (MF1)

The qualitative results not only confirmed the quantitative findings but also provided deeper insight into the patterns observed. A comparison of the quantitative and qualitative results regarding students’ motivations for studying nursing is presented in Table 6.

**Table 6:** Comparison between quantitative and qualitative findings on students’ motivation for studying nursing

Motivation Category	Scale Factor	Scale Item	Qualitative Subtheme	Qualitative Theme
Intrinsic motivation	Pursuit of Knowledge	Interested in the content of nursing courses	Interest in nursing	Choosing courses of interest
	Enthusiasm	From childhood, aspired to become a nurse	Belief that nursing embodies noble ideals	Nursing is a calling
	Personal Values	Desire to help others	Helping others	Desire for professional knowledge and helping others
	Professional values and skills	Want to help others and relieve their suffering		
		I want to learn correct nursing knowledge	Increasing medical knowledge	
Extrinsic motivation	Attainment of Status	To pass the professional certification exam	Gaining the title and professional status of a registered nurse	Obtaining a nursing license
	Career Advancement	To find a better job	Multiple employment opportunities	Job stability
		To increase job opportunities		
		To enhance work or employment ability		
		Nurses have stable income	Stable income	
		Nurses have stable jobs		

### Students’ perceptions of nursing

Students described nursing as a demanding profession that requires physical endurance, responsibility, and emotional sensitivity.

#### Busy

Participants noted that nursing work involves irregular schedules and heavy workloads.

##### Irregular day–night shifts

*The working hours are not fixed… sometimes in the morning*,* sometimes in the evening.* (HG1)

##### Exhausting work

*Some patients are in critical condition*,* which makes the work busier and more chaotic.* (UG2)

##### High stress tolerance required

*You need strong stress tolerance… it’s more stressful than most jobs.* (EG3)

#### Sense of responsibility

Students emphasized the importance of professional competence and attentiveness.

##### Professional and technical competence

*We must keep up with new knowledge and techniques to improve our professional competence.* (FH1)

##### Observing conditions and understanding needs

*You have to listen to patients and observe their conditions carefully.* (CH2)

#### Empathy

Empathy was regarded as a core attribute of nursing practice.

##### Being gentle and patient

*Nurses need to be gentle and patient to comfort anxious patients and families.* (FI1)

##### Being attentive

*You have to be organized and careful—you can’t forget important things.* (EI2)

Empathy was viewed as an essential personal attribute in nursing practice.

## Discussion

The finding that extrinsic motivation significantly predicted academic performance in *Introduction to Nursing*, but not in *First Aid* or overall semester GPA, may be explained by differences in course characteristics, as well as students’ stage of professional socialization.

*Introduction to Nursing* emphasizes professional orientation, role understanding, and future career imagination, which align closely with extrinsic motives such as obtaining a nursing license, social recognition, and career stability. In contrast, *First Aid* is a skill-oriented course that relies more heavily on hands-on practice and short-term performance, where motivational orientation may not yet be directly translated into measurable academic outcomes among first-year students. Rather than reflecting short-term learning engagement alone, the predictive role of extrinsic motivation in Introduction to Nursing may indicate that first-year students’ academic efforts are closely tied to future-oriented considerations, such as licensure, career stability, and social recognition. At this early stage of professional socialization, these externally regulated goals may function as concrete anchors that help adolescents navigate the abstract professional concepts introduced in foundational nursing courses.

Qualitative findings further suggested that students’ motivations were shaped by interest in nursing, altruistic values, family influence, and pragmatic considerations related to employment stability. Together, the quantitative and qualitative results highlight the coexistence of value-driven and outcome-oriented motivations during the early stage of nursing education.

The qualitative theme of “nursing as a calling” reflects a form of intrinsic motivation characterized by internalized values, personal meaning, and a sense of purpose in professional identity development. Although intrinsic motivation did not significantly predict academic performance in the quantitative analysis, this theme suggests that such motivation may play a more prominent role in shaping students’ long-term commitment to the profession rather than immediate academic outcomes. For first-year students in five-year junior college programs, intrinsic motivation may still be in an early developmental stage and may not yet be strongly translated into measurable academic performance.

Table 6 further illustrates points of convergence and divergence between the quantitative and qualitative findings.

Specifically, the qualitative themes related to career stability, professional recognition, and licensure converge with the quantitative finding that extrinsic motivation predicts performance in *Introduction to Nursing*. In contrast, the absence of corresponding qualitative emphasis on short-term skill performance may help explain the non-significant association observed for *First Aid*.

These interpretations should be viewed as exploratory and context-specific, as the present study was conducted in a single institutional setting with first-year nursing students. Future studies incorporating longitudinal designs or multi-institutional samples are warranted to further examine these relationships.

### Interpretation of qualitative findings on students’ motivation to study nursing

The qualitative findings of this study indicated that students’ motivation to study nursing was primarily driven by interest in nursing, family influence, employment opportunities, and the desire to help others using professional knowledge. These findings are consistent with existing literature on nursing students’ career choice and motivation.

Students reported choosing nursing because they were genuinely interested in the profession. This finding aligns with the study by Jirwe and Rudman (2012) [19], which demonstrated that a sincere interest in nursing is a key reason students decide to enter nursing programs. Such interest reflects an early engagement with the profession and suggests that students’ initial motivation is not purely instrumental but also value-oriented.

Family influence also emerged as an important motivating factor in students’ decision-making, with some students reporting that family members encouraged them to enroll in nursing programs. This result is consistent with findings by McKenna et al. (2023) [9], who reported that family influence was one of the key motivations for nursing students entering the profession.

In addition, students indicated that they chose nursing because of diverse employment opportunities and stable income. This finding is consistent with Sillero et al. (2023) [10], who reported that Generation Z nursing students seek better employment conditions, understood as a stable job and salary.

Furthermore, students expressed willingness to study nursing because they wished to help others through professional knowledge, while also valuing stable income and job security. These motivations closely correspond to the findings of Başkale and Serçekuş (2015) [20], who identified students’ desire to help others, satisfaction with income, and work stability as central reasons for choosing nursing. Together, these findings indicate that altruistic motives and economic considerations coexist in shaping students’ decisions to pursue nursing education.

### Interpretation of students’ perceptions of the nursing profession

Students in this study perceived nursing as a profession that requires high-level professional skills, continuous learning, a strong sense of responsibility, and empathy. Participants emphasized that nurses must constantly update their professional knowledge, carefully observe patients’ conditions, understand patients’ needs, and demonstrate patience and gentleness when providing care.

These perceptions are consistent with the findings reported by Cheng (2016) [21], who noted that nursing students commonly view nursing as a profession requiring ongoing improvement, innovation, intellectual competence, and careful, patient-centered care. Similarly, Andina-Díaz et al. (2023) [22] found that nursing students perceive nursing work as demanding proficiency in multiple tasks, attention to detail, and strong empathetic abilities. The alignment between the present findings and prior studies suggests that students develop a relatively realistic understanding of the professional demands of nursing even at an early stage of education.

### Interpretation of quantitative findings through motivation theory

The findings indicate that extrinsic motivation was a significant predictor of academic performance, particularly in Introduction to Nursing. In contrast, intrinsic motivation did not show a significant direct effect on any of the three academic outcomes.

According to Ryan and Deci (2000) [6], extrinsic motivation refers to engaging in an activity to attain separable outcomes, whereas intrinsic motivation involves performing an activity for its inherent satisfaction. The present findings suggest that first-year nursing students may rely more heavily on extrinsic motives—such as future career prospects and employment stability—when engaging with foundational nursing courses.

The qualitative findings showed that students expressed altruistic motives, such as wanting to help others by using professional knowledge. This finding is consistent with previous research indicating that altruism is a common motivation among newly admitted nursing students [14].

Intrinsic motivation is defined as an internal desire to engage in an activity [7]. In the present study, intrinsic motivation did not show a significant direct association with academic performance. In contrast, students’ emphasis on stable income and job security reflects extrinsic motivation, which is defined as behavior driven by anticipated rewards [7]. The strong predictive role of extrinsic motivation observed in this study suggests that externally regulated goals may be particularly influential during the early stage of nursing education.

### Educational implications and motivation development

Ryan and Deci (2000) [6] emphasized that although individuals possess an innate tendency toward intrinsic motivation, environmental conditions can either support or undermine its development. In the present study, students entered nursing programs with positive learning motivation and favorable perceptions of the profession. However, how intrinsic motivation is sustained during subsequent clinical training warrants further investigation.

Previous research has shown that excessive workload, burnout, and intentions to leave the profession among clinical nurses can negatively affect healthcare quality [23]. In the context of Taiwan’s busy clinical environments, students may experience a mismatch between their initial altruistic expectations and real-world working conditions, potentially weakening intrinsic motivation and reducing willingness to remain in the nursing workforce.

Developing nursing students’ professional identity has been identified as a key strategy for supporting their transition from student to professional nurse. Gilvari et al. (2022) [24] demonstrated that stronger professional identity enhances occupational commitment, and supportive learning environments have been shown to facilitate professional identity development [25].

Finally, resilience training may help healthcare students cope with study- and work-related stress. A Cochrane review reported very-low certainty evidence that, compared with controls, resilience training in healthcare students may reduce stress or stress perception and anxiety at post-intervention (Kunzler et al., 2020) [26]. Incorporating resilience-focused content into nursing education may therefore be beneficial for supporting students’ well-being during training.

In practice, motivation-oriented strategies may include incorporating structured career exploration activities into foundational nursing courses, such as inviting clinical nurses to share real-world experiences, discussing diverse nursing career pathways, or guiding students to reflect on their future professional roles. Career-related guidance may also involve early exposure to licensure requirements, employment conditions, and long-term career development opportunities to help students link course content with future professional goals.

Resilience training could be integrated into the existing curriculum through brief reflective exercises, stress-management workshops, or scenario-based discussions embedded within nursing courses. For example, guided reflection on academic stress, peer-support activities, or short modules on coping strategies and self-care could be incorporated without adding substantial curricular burden.

### Implications for nursing education

5. Enhancing Motivation and Professional Identity through Curriculum Design

Developing a strong professional identity helps nursing students transition into competent professionals and enhances their commitment to the profession [24]. Supportive learning environments and academic collaboration have been identified as crucial factors in fostering professional identity development among nursing students [25]. Accordingly, nursing schools should strive to create caring and supportive learning settings, both in classroom-based education and during clinical training.

Curriculum designs that promote early understanding of professional roles and career pathways may further support students’ motivation and professional identity formation. Integrating career-related content into first-year courses may help students develop a clearer sense of direction and strengthen their engagement with the nursing profession over time.

6. Developing Resilience-Building Courses

Resilience refers to the ability to maintain or regain psychological well-being when facing challenges. Nursing students encounter various stressors related to learning and clinical training, and resilience training has been shown to produce positive effects [26]. Nursing programs can incorporate resilience-focused courses—such as mindfulness-based interventions or cognitive-behavioral therapy—to improve students’ coping and stress-management skills.

### Quantitative–qualitative integration

The logistic regression results showed that extrinsic motivation was a significant predictor of students’ performance in *Introduction to Nursing*, but not for *First Aid* or overall semester grades. Intrinsic motivation did not significantly predict any of the three outcomes. Both the t-test and regression analyses demonstrated that extrinsic motivation influenced achievement in *Introduction to Nursing*.

A possible explanation is that students may have already developed a strong identification with the nursing profession prior to enrollment, which enhanced their engagement with *Introduction to Nursing*. In contrast, *First Aid* is a more practice-oriented course; first-year students may still be in the adaptation phase, so motivation levels might not yet align with performance. Furthermore, since first-year students take only two nursing-related courses, while other subjects are general education, motivation may not significantly predict overall semester results.

Given that extrinsic motivation affected students’ performance in *Introduction to Nursing*, educators should pay attention to those performing poorly in this subject. Integrating teaching strategies that enhance understanding of nursing work environments and career pathways may help strengthen students’ extrinsic motivation and engagement.

This suggests that external incentives, such as future career stability and financial security, may drive students’ engagement with foundational nursing courses.

The participants in this study were primarily aged 15–16 years and enrolled in a five-year junior college nursing program, which differs from the educational trajectories of older nursing students in four-year BSN programs. Compared with older students, who may be more influenced by autonomous career choice or prior educational experiences, younger students at this early stage may place greater emphasis on externally oriented motivations, such as job security and social expectations. Therefore, caution is warranted when extending these findings to nursing students in different age groups or educational systems.

## Conclusion

Establishing supportive and positive educational and clinical learning environments may help sustain nursing students’ altruism and motivation to serve others during their training. By fostering professional identity and balanced motivational orientations in the early stage of nursing education, these findings may offer insights for nursing education programs in different contexts that face challenges in supporting student engagement and professional development.

### Limitations

This study was limited to a sample of first-year students enrolled in a five-year junior college nursing program at a university of science and technology in southern Taiwan. A total of 93 valid questionnaires were collected, and 24 students participated in focus group interviews. Because of the relatively small and regionally restricted sample, the external validity of the findings is limited, and the results may not be generalizable to nursing students from other regions, educational programs (such as four-year or two-year technical programs), or different academic years.

In southern Taiwan, perceived advantages such as stable income and job security may be particularly salient due to local labor-market conditions and family expectations. This contextual factor may have amplified the qualitative emphasis on “job stability” observed in this study. Future studies are recommended to increase the sample size and expand geographical coverage to enhance the generalizability of the findings.

In addition, this study focused only on the relationship between learning motivation and academic performance and did not explore potential long-term relationships with career planning or professional identity development. Future research could adopt longitudinal designs to examine how value-based education integrated into nursing curricula may promote professional identity formation and long-term career development among nursing students.

## Supplementary Information

Below is the link to the electronic supplementary material.

Supplementary Material 1

## Data Availability

The datasets generated and/or analyzed during the current study are available from the corresponding author on reasonable request.

## Abbreviations

DMSN: Diverse motivations for studying nursing
SDT: Self-determination theory
GPA: Grade point average
IRB: Institutional review board
