# Supplementary material for: Study motivation and academic performance among first-year five-year junior college nursing students: a mixed-methods study
Source: BMC Nurs. 2026 Mar 2;25:320. doi: 10.1186/s12912-026-04460-y (PMC13059224; doi:10.1186/s12912-026-04460-y)
Supplement: Supplementary file 1 — Supplementary Material 1 [file 12912_2026_4460_MOESM1_ESM.docx]

**Additional file 1. Semi-structured interview guide (English translation)**

This supplementary file presents the English translation of the semi-structured interview guide used in the qualitative phase of the study. The guide was designed to explore first-year junior college nursing students’ motivations for studying nursing and their perceptions of the nursing profession.

- Guiding questions:

1. Why did you choose to study nursing?

2. What are your perceptions of nursing work?

Note: The interviews were conducted in Mandarin Chinese, and this English translation was prepared for submission as a supplementary file to ensure transparency and replicability.
